# Myocardial dysfunction occurs prior to changes in ventricular geometry in mice with chronic kidney disease (CKD)

**DOI:** 10.14814/phy2.12732

**Published:** 2016-03-20

**Authors:** Pamela D. Winterberg, Rong Jiang, Josh T. Maxwell, Bo Wang, Mary B. Wagner

**Affiliations:** ^1^ Division of Pediatric NephrologyDepartment of PediatricsEmory University School of MedicineAtlantaGeorgia; ^2^ Children's Heart Research & Outcomes (HeRO) CenterChildren's Healthcare of Atlanta & Emory UniversityAtlantaGeorgia; ^3^ Division of Pediatric CardiologyDepartment of PediatricsEmory University School of MedicineAtlantaGeorgia; ^4^ Wallace H Coulter Department of Biomedical EngineeringEmory University School of MedicineAtlantaGeorgia

**Keywords:** Animal model, experimental, blood pressure, cardiomyopathy, chronic kidney failure, echocardiography, fibrosis, endomyocardial, mice, uremia, ventricular dysfunction

## Abstract

Uremic cardiomyopathy is responsible for high morbidity and mortality rates among patients with chronic kidney disease (CKD), but the underlying mechanisms contributing to this complex phenotype are incompletely understood. Myocardial deformation analyses (ventricular strain) of patients with mild CKD have recently been reported to predict adverse clinical outcome. We aimed to determine if early myocardial dysfunction in a mouse model of CKD could be detected using ventricular strain analyses. CKD was induced in 5‐week‐old male 129X1/SvJ mice through partial nephrectomy (5/6Nx) with age‐matched mice undergoing bilateral sham surgeries serving as controls. Serial transthoracic echocardiography was performed over 16 weeks following induction of CKD. Invasive hemodynamic measurements were performed at 8 weeks. Gene expression and histology was performed on hearts at 8 and 16 weeks. CKD mice developed decreased longitudinal strain (−25 ± 4.2% vs. −29 ± 2.3%; *P* = 0.01) and diastolic dysfunction (E/A ratio 1.2 ± 0.15 vs. 1.9 ± 0.18; *P* < 0.001) compared to controls as early as 2 weeks following 5/6Nx. In contrast, ventricular hypertrophy was not apparent until 4 weeks. Hearts from CKD mice developed progressive fibrosis at 8 and 16 weeks with gene signatures suggestive of evolving heart failure with elevated expression of natriuretic peptides. Uremic cardiomyopathy in this model is characterized by early myocardial dysfunction which preceded observable changes in ventricular geometry. The model ultimately resulted in myocardial fibrosis and increased expression of natriuretic peptides suggestive of progressive heart failure.

## Introduction

Patients with chronic kidney disease (CKD) have an estimated 10‐ to 100‐fold increased risk of cardiovascular mortality than their peers (Groothoff et al. 2002; Parekh et al. 2002; Tonelli et al. 2006; Hallan et al. 2012). Clinical trials of therapies targeting traditional (Palmer et al. 2013) and CKD‐specific (Eknoyan et al. 2002; Drüeke et al. 2006; Zannad et al. 2006; Jamison et al. 2007; Locatelli et al. 2010) cardiovascular risk factors have failed to significantly improve morbidity and mortality in patients with advanced‐stage CKD. Uremic cardiomyopathy (Groothoff et al. 2002; Parekh et al. 2002) is a significant cause of morbidity and mortality among patients with CKD, but the underlying mechanisms contributing to this complex phenotype are incompletely understood (Stenvinkel et al. 2008; Alhaj et al. 2013).

Left ventricular hypertrophy (LVH) and diastolic dysfunction are recognized features of uremic cardiomyopathy in adults (Hayashi et al. 2006; Edwards et al. 2008a; Park et al. 2012; Asp et al. 2015) and children (Mitsnefes et al. 2006; Matteucci et al. 2013; Scavarda et al. 2014) with CKD. Indices of diastolic dysfunction are associated with LVH in patients with CKD (Mitsnefes et al. 2004; Hayashi et al. 2006), supporting the hypothesis that hypertrophy contributes to the myocardial stiffness of uremic cardiomyopathy. Intriguingly, evidence suggests that diastolic dysfunction may precede the development of LVH in patients (Aeschbacher et al. 2001; Di Bello et al. 2010) and rats (Dupont et al. 2012) with essential hypertension. These recent observations raise interesting questions about the temporal and mechanistic relationships of diastolic dysfunction, LVH, and systemic hypertension during CKD and underscore the importance of defining these relationships in common animal models used to interrogate the underlying mechanisms of uremic cardiomyopathy. A classical rat model of CKD, the 5/6 nephrectomy model (5/6Nx), has been adapted for use in mice in recent years (Edwards et al. 2008a; Chinali et al. 2015). Several strains of mice undergoing 5/6Nx have been demonstrated to develop cardiac hypertrophy and diastolic dysfunction (Kennedy et al. 2008; Siedlecki et al. 2009; Lin et al. 2015), however, there is limited information about the kinetics of myocardial dysfunction and structural changes during this model of CKD.

Advancements in imaging technology are enabling earlier detection of subclinical cardiac dysfunction in patients and animal models. One such example is strain‐based imaging, a noninvasive echocardiographic technique which tracks segmental and global myocardial deformation with the capability of differentiating between active and passive movement of the myocardium (Dandel et al. 2009). Decreased ventricular strain is considered a sensitive measure of myocardial dysfunction and predicts mortality in patients with cardiomyopathy, congestive heart failure, and myocardial infarction (Roes et al. 2009; Haugaa et al. 2013; Tee et al. 2013). Recent reports using strain‐based imaging modalities detected subclinical myocardial dysfunction in patients with early‐stage CKD (Nasir et al. 2007; Chinali et al. 2015) which correlated with cardiovascular events and mortality (Rakhit et al. 2007; Edwards et al. 2008b). Therefore, understanding and targeting the early pathogenic factors contributing to subclinical myocardial dysfunction may be necessary to improve cardiovascular morbidity and mortality for patients with CKD. The use of strain‐based imaging in small animal models has only recently been achievable with the availability of microultrasound systems with sufficiently high resolution and frame rate needed to analyze small, rapidly beating rodent hearts (Koshizuka et al. 2013). Consequently, there is limited literature on strain‐based imaging techniques in mouse models of cardiovascular disease (Bauer et al. 2011; Andrews et al. 2014; Bhan et al. 2014) and as of yet no report of its use in models of uremic cardiomyopathy.

Myocardial dysfunction during uremic cardiomyopathy is best characterized as diastolic dysfunction with preserved ejection fraction. Diastolic dysfunction can occur as a result of impaired myocardial active relaxation or increased ventricular passive stiffness. Myocardial relaxation is mediated by alterations in cardiomyocyte calcium transients, myofilament sensitivity to calcium, and cellular energetics (Belke and Dillmann 2004; Borbély et al. 2005; Davis et al. 2012; Abdurrachim et al. 2014). Under normal physiological conditions, cytosolic calcium concentration [iCa^2+^] rapidly increases during systole to facilitate myofilament contraction, and subsequently sequestered into the sarcoplasmic reticulum (SR) during diastole to allow myofilament relaxation. Reuptake of iCa^2+^ into the SR during diastole occurs primarily via the SR calcium ATPase (SERCA2a). SERCA2a dysfunction resulting in accumulation of iCa^2+^ during diastole and consequently incomplete myofilament relaxation, has been implicated in the impaired relaxation of cardiomyocytes (CM) isolated from failing hearts in humans (Beuckelmann et al. 1992, 1995) and animals (Bing et al. 1991; Krüger et al. 1994; Yao et al. 1998). Cardiomyocytes isolated from the hearts of uremic rats have been reported to display accumulation of diastolic calcium with prolonged time constant for calcium reuptake correlating with reduced SERCA2a expression, protein density, and activity (McMahon et al. 2002; Kennedy et al. 2003). SERCA2a expression was decreased in the hearts of CD‐1 mice following partial nephrectomy (Kennedy et al. 2008), however, we can find no reports of calcium handling in the mouse model of uremic cardiomyopathy.

Using a common inbred mouse strain previously reported in the literature to develop uremic cardiomyopathy (Siedlecki et al. 2009), we set out to determine (1) the temporal development of myocardial dysfunction and hypertrophy, (2) whether early myocardial dysfunction could be detected using speckle‐tracking strain analysis, and (3) whether myocardial fibrosis or changes in myocyte calcium handling could explain impaired myocardial relaxation in uremic cardiomyopathy.

## Materials and Methods

### CKD model

Male 129X1/SvJ mice (The Jackson Laboratory, Bar Harbor, ME) age 5–6 weeks were randomly assigned to undergo five‐sixth nephrectomy (5/6Nx) surgery (*n* = 14) or sham surgeries (*n* = 11) under inhaled isoflurane (2%) anesthesia, in a two‐stage approach. In the first stage, the left kidney was exposed via flank incision and decapsulated to avoid ureter and adrenal damage. The upper and lower poles of the left kidney were resected via selective cauterization with a high‐temperature fine tip cautery (Geiger Medical Technologies, Council Bluffs, IA). After 1 week recovery, the entire right kidney was removed via a right flank incision. Sham surgeries involved flank incision, exposure of kidneys, but no removal of tissue at the same timing as 5/6Nx surgeries. Mice were fed 2018 Teklad Global 18% Protein Rodent Diet (Envigo, Madison, WI) ad libitum. All animal experiments were conducted in accordance with the National Institutes of Health *Guide for the Care and Use of Laboratory Animals* using protocols approved by Emory University Institutional Animal Care and Use Committee. Renal function was assessed by measuring urea nitrogen concentration via colorimetric assay (Arbor Assays, Ann Arbor, MI) and Cystatin c concentration using an ELISA assay (R&D Systems, Minneapolis, MN) in plasma samples.

### Echocardiography

**Figure 1:**
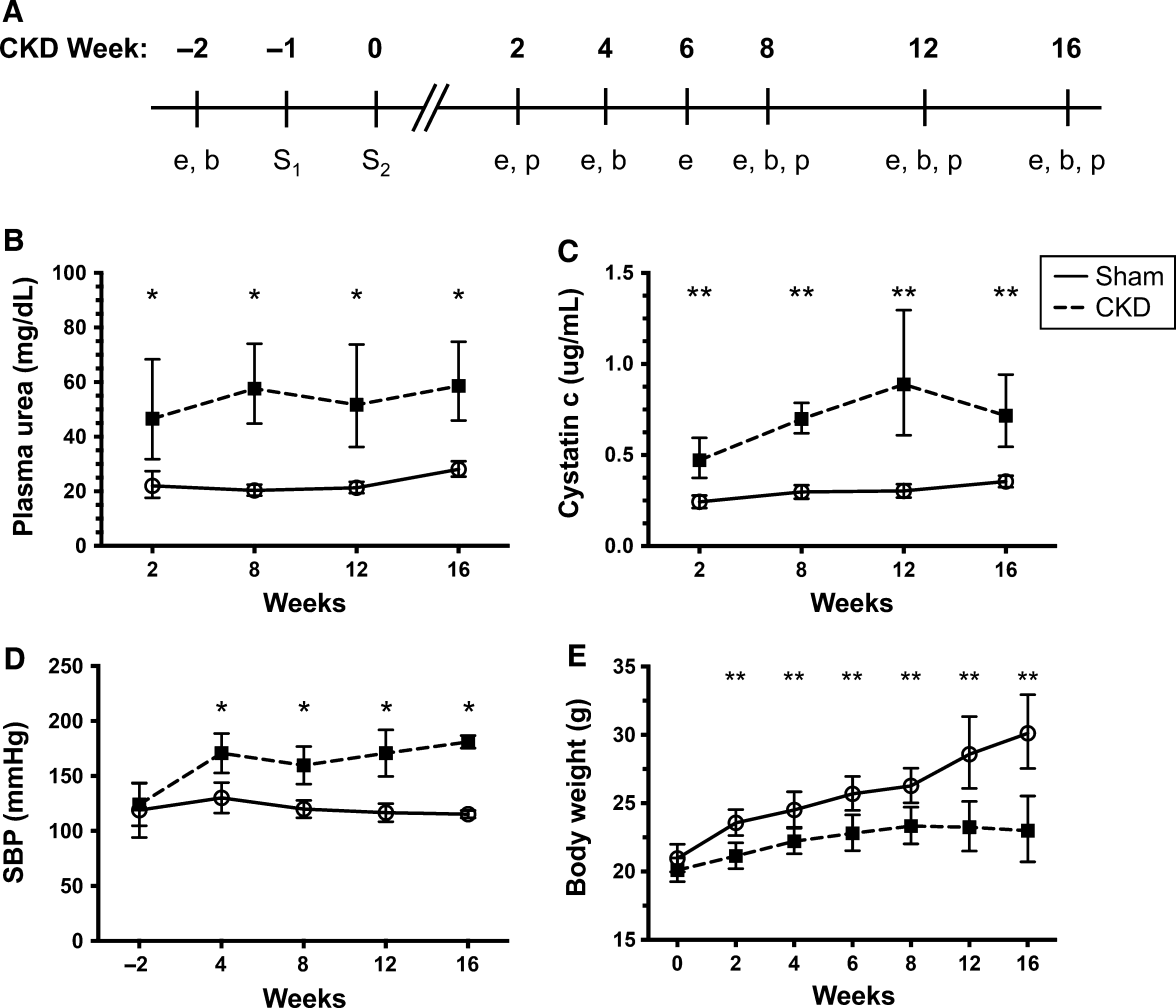
Partial nephrectomy (5/6Nx) results in chronic uremia and hypertension. Experimental design is presented in (A) where S_1_ represents surgery 1, S_2_ surgery 2, “e” echocardiography, “b” blood pressure measurement, “p” plasma studies. Trends in kidney function (plasma urea [B] and cystatin c [C]), systolic blood pressure (D), and body weight (E) in Sham versus chronic kidney disease (CKD) mice. *P < 0.05, **P < 0.01 between Sham and CKD groups at the same time point.

Echocardiographic studies were performed at baseline (prior to surgeries), then every 2 weeks through 8 weeks of CKD, and every 4 weeks thereafter until 16 weeks (see Fig. 1A). Mice were lightly anesthetized with 1–2% isoflurane/100% oxygen and placed on a warming platform set to 37°C for the duration of the recordings. The heart rate was monitored simultaneously by electrocardiography and maintained at 450–500 beats per minute. Cardiac image sequences were acquired using a Vevo 2100 digital high‐frequency ultrasound system (FujiFilm Visualsonics Inc, Toronto, ON, Canada) equipped with a probe (MS400, 30‐MHz) suited for mouse imaging. Standard 2D echocardiographic measurements of left ventricular (LV) dimensions were performed in the short‐axis view. LV volumes and LV mass were estimated from traced images in the parasternal long‐axis view. Relative wall thickness (RWT) was calculated as (2 × LVAWd)/LVIDd.

Mitral valve flow Doppler was acquired in an apical four‐chamber view. LV diastolic function was assessed by measuring the wave ratio of the LV transmitral early peak flow velocity to LV transmitral late peak flow velocity (the E/A ratio). M‐mode and Doppler measurement data represent 4–5 averaged cardiac cycles from at least two scans per mouse.

Strain analyses were conducted by the same trained investigator (RJ) for all images using speckle tracking software, Vevostrain^TM^ Analysis (FujiFilm Visualsonics, Inc, Toronto, ON, Canada). Global strain measurements in the longitudinal and radial directions were quantified using B mode cine images in the LV parasternal long‐axis view. All strain data were measured and averaged over at least three heart beats.

### Noninvasive blood pressure measurement

Blood pressures (BP) were measured using noninvasive tail‐cuff measurements (BP‐2000 Blood Pressure Analysis System, Visitech Systems, Apex, NC) at baseline and every 4 weeks until endpoint (Fig. 1A). BP measurements from the third consecutive day were recorded and used for analysis to account for behavioral acclimation.

### Invasive hemodynamics

Invasive hemodynamic measurements were performed at 8 weeks following surgery in a separate cohort of mice. Mice were anesthetized with inhaled isoflurane, intubated and ventilated using a small animal volume‐controlled ventilator (Inspira ASV, Harvard Apparatus, Holliston, MA). A 1F pressure‐volume conductance catheter (Millar, Inc., Houston, TX) was inserted into the left ventricle via apical puncture. Data were analyzed using LabChart analysis software (v7, ADInstruments, Colorado Springs, CO).

### Histology

Hearts were removed, flushed with phosphate‐buffered saline, submerged in 100 mmol/L KCl to arrest in diastole, then fixed in 10% buffered formalin. Fibrosis quantification was performed in ImageJ (Schneider et al. 2012) using 20× magnification images of digitized (Hamamatsu Nanozoomer 2.0HT) PicoSirius stained paraffin‐embedded sections of 5 *μ*m thickness.

### qRT‐PCR

Left ventricles were dissected from excised hearts and preserved in RNAlater^TM^ (Life Technologies, Carlsbad, CA). RNA was isolated from ventricular homogenates using miRNeasy mini kit (Qiagen, Frederick, MD). Complementary DNA was generated using High Capacity cDNA Reverse Transcription Kit (ThermoFisher Scientific, Waltham, MA) according to manufacturers’ instructions. See Table 1 for list of TaqMan primer‐probe sets (ThermoFisher Scientific) run on the StepOnePlus^TM^ real‐time PCR system (ThermoFisher Scientific). Relative gene expression was calculated using the ΔΔCt method normalized to the housekeeping gene Rn18s, and is presented as fold change compared to expression in Sham samples at each time point.

**Table 1:** Primers used in qRT‐PCR experiments

Gene symbol	Official gene name	Other names	NCBI gene reference	TaqMan assay ID
Atp2a2	ATPase, Ca^++^ transporting, cardiac muscle, slow twitch 2	SERCA2a	NM_001110140.3	Mm01201431_m1
Slc8a1	Solute carrier family 8 (sodium/calcium exchanger), member 1	NCX	NM_001112798.2	Mm01232254_m1
Pln	Phospholamban		NM_001141927.1	Mm04206542_m1
Ryr2	Ryanodine receptor 2, cardiac		NM_023868.2	Mm00465877_m1
Nppa	Natriuretic peptide type A	ANP	NM_008725.2	Mm01255747_g1
Nppb	Natriuretic peptide type B	BNP	NM_008726.4	Mm01255770_g1
Col1a1	Collagen, type I, alpha 1		NM_007742.3	Mm00801666_g1
Ctgf	Connective tissue growth factor	Ccn2	NM_010217.2	Mm01192932_g1
Rn18s	18s Ribosomal RNA		NR_003278.3	Mm03928990_g1

### Calcium transients of isolated CM

Mouse ventricular myocytes were isolated by enzymatic dissociation. The hearts of anesthetized mice were excised and perfused with Krebs‐Ringer solution followed by an enzymatic solution (Worthington Type II) for 17–25 min. The left ventricle was dissected and placed into a KB (storage) solution. The tissue was then minced and triturated to separate individual myocytes and filtered to remove large, undissociated fragments. Sarcomere shortening and calcium transients were measured as we have previously described (Chen et al. 2012). Briefly, isolated myocytes were loaded with 2–5 *μ*mol/L Fura‐2AM (ThermoFisher Scientific) for 20 min at room temperature then placed in the stimulation chamber and perfused with Tyrode Buffer for 30 min to allow for de‐esterification of the dye. Cells were paced by field stimulation at 0.5, 1, and 2 Hz, and imaged with a dual‐excitation fluorescence photomultiplier system (IonOptix, Milton, MA) allowing for simultaneous recording of sarcomere length and Fura‐2 emission ratio throughout each beat. Calcium transients were analyzed using IonWizard software (v6, IonOptix).

### Statistics

Unpaired, two‐tailed *t*‐tests were performed with pooled variances (Prism v6.0c, GraphPad Software, Inc, La Jolla, CA) for data with normal distributions. Nonparametric tests (Mann–Whitney) were performed for data that did not have a normal distribution. Data with normal distribution are presented as means with standard deviation, and those with non‐normal distribution are graphically presented as geometric means with 95% confidence intervals. A *P*‐value < 0.05 was considered statistically significant.

## Results

### Partial nephrectomy results in chronic kidney disease

We first verified CKD induction via the partial nephrectomy model (5/6Nx) in the 129X1/SvJ mouse strain. Mice undergoing 5/6Nx developed elevated plasma urea nitrogen levels at 2 weeks following surgery (48.6 ± 17.8 vs. 22.1 ± 3.0 mg/dL; *P* = 0.02) that persisted throughout the study (61.6 ± 23.4 vs. 28.2 ± 3.2 mg/dL at 16 weeks; *P* = 0.002) consistent with CKD. Cystatin‐c levels were also elevated in mice undergoing 5/6Nx compared to sham controls at 2 weeks (0.48 ± 0.07 vs. 0.24 ± 0.07 *μ*g/mL; *P* = 0.003) and remained elevated throughout the study. Systolic BP were increased compared to controls and remained stable throughout the 16‐week study (Fig. 1). In summary, we confirmed results previously reported in the literature (Ma and Fogo 2003; Siedlecki et al. 2009; Leelahavanichkul et al. 2010) that partial nephrectomy in 129X1/SvJ mice results in a clinical phenotype similar to patients with CKD.

### Ventricular dysfunction precedes changes in ventricular geometry

**Figure 2:**
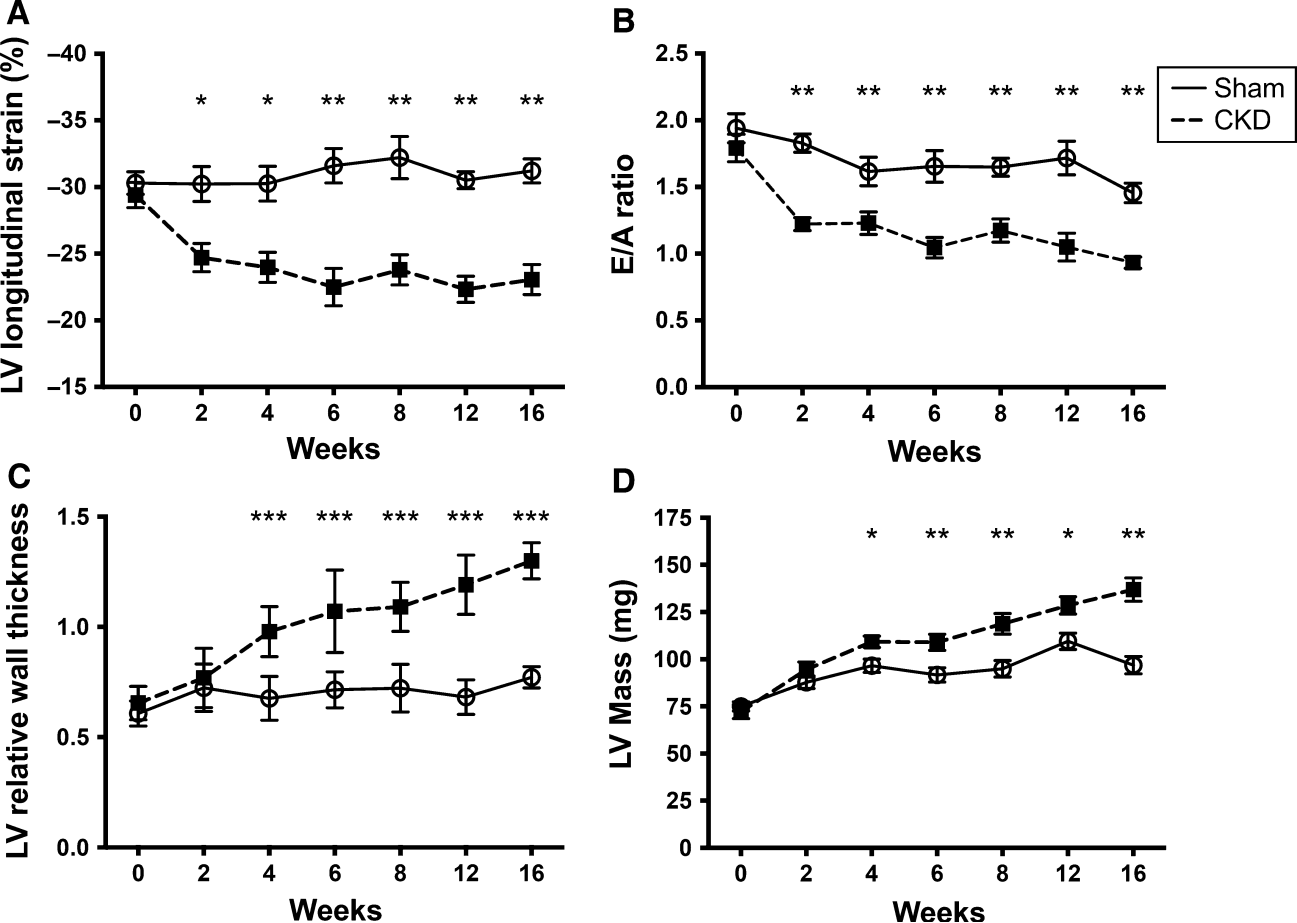
Temporal course of left ventricle structural and functional changes in chronic kidney disease (CKD) mice as measured by longitudinal echocardiography. Functional changes include global longitudinal strain (A) and mitral flow E/A ratio (B). Measures of structural remodeling include relative wall thickness (C) and left ventricular (LV) mass [median with 95% CI] (D) over time. Mean and standard deviations presented unless otherwise indicated; *indicates *P* < 0.05, **P < 0.01, ***indicates *P* < 0.001 between Sham and CKD groups at the same time point.

We next set out to determine the temporal kinetics of myocardial dysfunction and hypertrophy in CKD mice using longitudinal echocardiographic measurements. Interestingly, myocardial deformation, assessed by global longitudinal strain, was significantly altered in CKD mice at the first measurement following CKD induction (2 weeks) and remained so over the course of the experiment (Fig. 2A). As shown in Figure 2B, transmitral flow velocity index (E/A ratio) was significantly lower in CKD mice (1.22 ± 0.15 vs. 1.88 ± 0.18; *P* < 0.001) early (2 weeks) following partial nephrectomy and progressed to reversal (ratio < 1) indicating severe diastolic dysfunction by 16 weeks of CKD (0.92 ± 0.11 vs. 1.43 ± 0.14; *P* < 0.001). LV mass (72.2 ± 11.4 vs. 74.9 ± 6.5 mg; *P* = 0.58), anterior wall thickness (0.99 ± 0.12 vs. 0.95 ± 0.05 mm; *P* = 0.49), and RWT (0.66 ± 0.08 vs. 0.61 ± 0.06; *P* = 0.42) were not significantly different between the groups at baseline. Surprisingly, LV remodeling was first detectable (4 weeks) after the development of ventricular dysfunction based on absolute anterior wall thickness (LVAW;d 1.39 ± 0.12 vs. 1.11 ± 0.09 mm; *P* < 0.001), RWT (Fig. 2C), and corrected LV mass (Fig. 2D). These results show that changes in ventricular function were detectable prior to commonly measured structural changes in the CKD model.

### CKD hearts have signs of diastolic dysfunction with preserved ejection fraction

**Figure 3:**
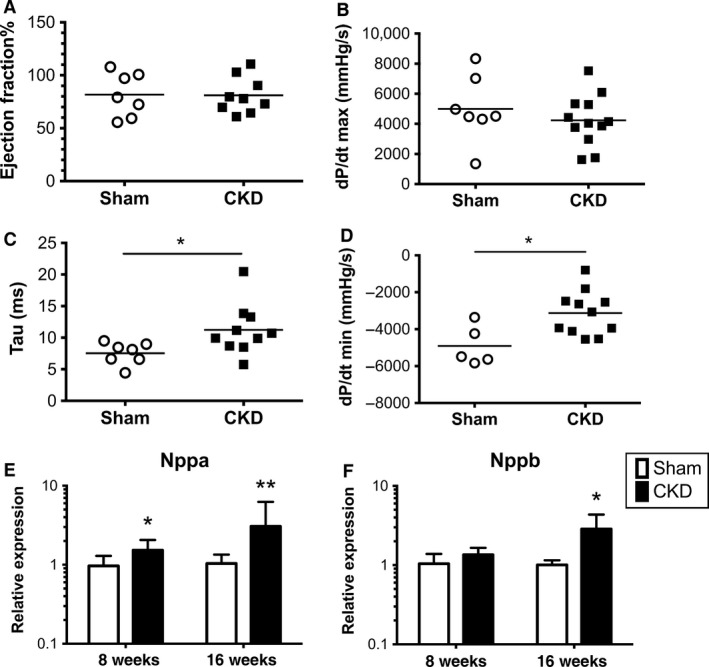
Chronic kidney disease (CKD) mice develop diastolic dysfunction with preserved ejection fraction. Invasive hemodynamic measures show preserved systolic function with no change in ejection fraction (A) or the rate of pressure increase during systole, dP/dt_Max_ (B), but signs of impaired relaxation as evidenced by prolonged relaxation time, Tau (C) and decreased rate of pressure decrease during diastole, dP/dt_Min_ (D). Hearts from CKD mice have increased expression of natriuretic peptides type A (Nppa, E) and type B (Nppb, F) compared to sham‐operated mice. *Indicates *P* < 0.05, **P < 0.01 between Sham and CKD groups at the same time point.

To evaluate the functional significance of the altered myocardial deformation seen on echo, we next determined ventricular systolic and diastolic function of the CKD mice via cardiac catheterization. Invasive hemodynamics at 8 weeks demonstrated impaired ventricular relaxation with significantly increased relaxation time constant (Tau) in CKD mice (13.3, IQR 10.3–17.2 vs. 8.7, IQR 7.1–9.4 ms; *P* = 0.016 Mann–Whitney). Consistent with this finding, the rate of ventricular relaxation (dP/dt_min_) was significantly lower in CKD mice (−3127 ± 362 vs. −4911 ± 478 mmHg/s; *P* = 0.02). Neither ejection fraction nor the rate of LV pressure rise during systole (dP/dt_max_) was significantly different between the groups (see Fig. 3). Calculated ejection fraction and fractional shortening on echo also did not significantly differ between the groups over the 16‐week time course (data not shown). Expression of A‐type natriuretic peptide (Nppa) was increased at 8 weeks and B‐type natriuretic peptide (Nppb) transcripts were increased at 16 weeks in heart tissue of CKD mice (Fig. 3E–F). Taken together, mice with CKD develop diastolic heart failure with preserved ejection fraction (HFpEF).

### CKD mice develop cardiac fibrosis

**Figure 4:**
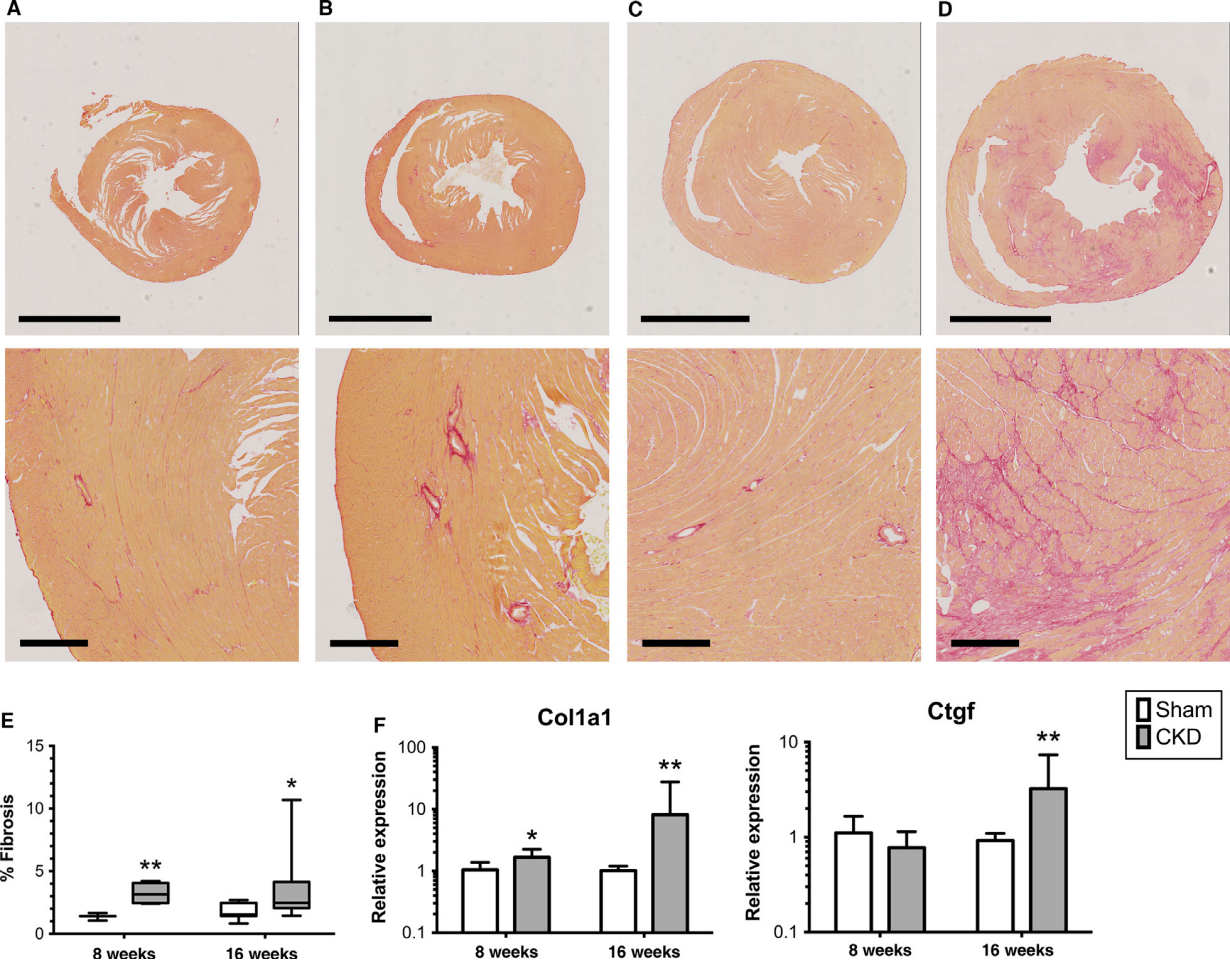
Chronic kidney disease (CKD) mice develop cardiac fibrosis. Representative photomicrographs of Picosirius red‐stained hearts from Sham at 8 weeks (A), CKD at 8 weeks (B), Sham at 16 weeks (C), and CKD at 16 weeks (D). Upper panel represents low‐power view (2× magnification, scale bar represents 2 mm) and bottom panel represents 10× magnification view of each picture above (scale bar represents 200 *μ*m). Fibrosis quantification is presented in (E). Relative expression of fibrosis‐related transcripts (F), type 1 collagen (Col1a1), and connective tissue growth factor (Ctgf). *P < 0.05, **P < 0.01 between Sham and CKD groups at the same time‐point.

Ventricular stiffness can contribute to impaired ventricular relaxation (Collier et al. 2011; Cheng et al. 2013), therefore, we assessed for cardiac fibrosis in our model. After 8 weeks, CKD mice have increased cardiac fibrosis on histology as detected by Sirius Red staining and gene signature of collagen remodeling with increased relative expression of type 1 collagen (Col1a1) and connective tissue growth factor (Ctgf) compared to sham controls (Fig. 4).

### Cardiomyocytes isolated from CKD mice do not have accumulation of diastolic calcium

**Figure 5:**
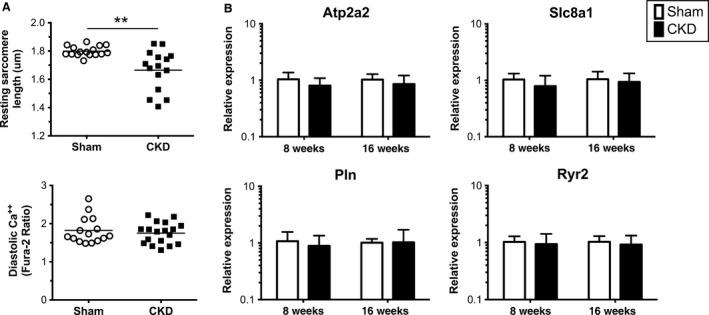
Calcium handling in isolated myocytes. Cardiomyocytes isolated from chronic kidney disease (CKD) mice at 8 weeks have shorter resting sarcomere length (A upper) but no appreciable accumulation of diastolic calcium (A lower). Measurements are from individual cells isolated from 3 to 4 mice per group are presented and are reflective of three separate experiments with comparative results. Hearts were collected at 8 and 16 weeks following surgery (*n* = 7–10 per group per time‐point) and mRNA expression quantified using qRT‐PCR for genes encoding proteins involved in sarcolemmal calcium handling (B). Atp2a2 (sarcoplasmic reticulum Ca^2+^ ATPase, SERCA2a), Slc8a1 (sodium/calcium exchanger, NCX), Pln (phospholamban), and Ryr2 (ryanodine receptor 2). **P < 0.01 between Sham and CKD.

SERCA2a dysfunction resulting in accumulation of diastolic calcium has been implicated in diastolic dysfunction (McMahon et al. 2002; Kennedy et al. 2003; Lacombe et al. 2007). Therefore, we examined measures of calcium handling in isolated CM at 4 and 8 weeks after CKD induction. Resting (diastolic) sarcomere lengths were not significantly different between CM from CKD mice compared to sham mice at 4 weeks (1.718, IQR 1.639–1.782 vs. 1.753, IQR 1.703–1.794 *μ*m; *P* = 0.32 Mann–Whitney), but were significantly shorter in cells from mice at 8 weeks of CKD (1.695, IQR 1.528–1.765 vs. 1.783, IQR 1.773–1.827 *μ*m; *P* = 0.008 Mann–Whitney). Despite the shorter diastolic sarcomere length, CMs isolated from CKD mice at 8 weeks did not display accumulation of diastolic calcium (Fig. 5A). No significant difference was found in calcium and sarcomere amplitudes, time to peak, or relaxation time (data not shown). Consistent with the results from isolated cells, relative expression of genes involved in calcium handling during excitation‐contraction cycling (Sarco[endoplasmic] reticulum Ca^2+^ ATPase [SERCA2a], Ryanodine receptor, Sodium calcium exchanger [NCX], and Phospholamban) was not significantly altered in the hearts collected at 8 or 16 weeks (Fig. 5B). In sum, we found no evidence to support a role for altered calcium cycling in this model of uremic cardiomyopathy.

## Discussion/Conclusions

This study presents a detailed examination of the longitudinal changes in cardiac structure and function in an experimental model of CKD. Uremic cardiomyopathy in this model is characterized by early myocardial dysfunction as demonstrated by altered indices of ventricular relaxation and myocardial deformation, which preceded observable changes in ventricular geometry. The model ultimately resulted in myocardial fibrosis and increased expression of natriuretic peptides suggestive of progressive heart failure. To our knowledge, this is the first published report of myocardial deformation analysis in a mouse model of uremic cardiomyopathy.

Ventricular stiffness has classically been considered a direct consequence of ventricular hypertrophy, so we were surprised to see impaired diastolic function as the earliest detectable change during uremic cardiomyopathy. Diastolic dysfunction has recently been noted to precede the development of LVH and elevation in BP in young adults with genetic predisposition for hypertension (Aeschbacher et al. 2001), adults with prehypertension (Di Bello et al. 2010), and in animal models (Dupont et al. 2012) of genetic hypertension. Dupont et al. (2012) described early onset of diastolic and systolic dysfunction prior to ventricular hypertrophy or BP elevation in the spontaneously hypertensive rat model. Koshizuka et al. (2013) showed that longitudinal strain impairment predicted progressive HFpEF in the Dahl salt‐sensitive rat model. Impaired myocardial deformation (as measured by longitudinal strain) was also seen prior to LVH in adults with prehypertension and hypertension and was associated with diastolic dysfunction (Di Bello et al. 2010). In our study, we do not have BP measurements prior to the first post‐CKD echo at 2 weeks, and are therefore unable to determine whether the functional changes preceded hypertension. Kennedy et al. (2008) described concurrent diastolic dysfunction and LVH in the partial nephrectomy model using the outbred CD‐1 mouse strain, however, the earliest time point reported for functional and structural measures was at 4 weeks following nephrectomy. It is unclear if myocardial dysfunction occurred prior to this first reported time point or if genetic variation between mouse strains could account for this difference.

It remains unclear if the altered myocardial deformation detected by ventricular strain analysis is directly related to LVH or diastolic dysfunction (Kasner et al. 2010). In our study, impaired longitudinal strain occurred simultaneously with impairment in both invasive and noninvasive measures of relaxation. Further study into the relationship between decreased ventricular strain on echocardiogram and diastolic dysfunction is needed.

Impaired myocyte calcium handling and consequent accumulation of cytosolic calcium during diastole have been implicated in the pathogenesis of early diastolic dysfunction in models of diabetic cardiomyopathy (Lacombe et al. 2007) and hypertensive LVH (Dupont et al. 2012). Kennedy et al. (2003), reported alteration in calcium cycling and contractile function in CM isolated from Sprague–Dawley rats undergoing 5/6Nx. However, we found no appreciable accumulation of calcium during diastole to account for the impaired relaxation of uremic mouse hearts at time points where diastolic dysfunction was detected both via invasive and noninvasive means. SERCA2a gene expression was also reported to be decreased in the hearts of CD‐1 mice as early as 4 weeks following partial nephrectomy (Kennedy et al. 2008), however, we found no significant difference in the expression of four genes encoding the major proteins regulating cardiomyocyte calcium handling between CKD and sham‐operated mice in our study. We have found no other reports of direct measurements of calcium handling in CM from mice with CKD using modern techniques and equipment. Our data underscore the importance of genetic background contributing to variation in underlying pathophysiology of the same CKD model in different mouse strains. While both strains, 129X1/SvJ and CD‐1, develop diastolic dysfunction, they appear to have differing underlying etiologies or pathways leading to this common functional outcome and therefore may have disparate responses to various therapeutic interventions.

Our model ultimately resulted in cardiac fibrosis and expression of BNP, which are associated with progressive heart failure and likely represent more “permanent” changes in cardiac structure and ventricular dysfunction. Determining the precise point at which compensatory hypertrophy converts to heart failure remains a challenge in the field of cardiovascular biology and is beyond the scope of this report.

Similar to our model, impaired myocardial deformation has been detected prior to the classical measures of systolic function in patients with mild CKD (Nasir et al. 2007; Edwards et al. 2008b). The fact that subclinical myocardial dysfunction associated with mortality (Rakhit et al. 2007; Edwards et al. 2008b) in early CKD and that clinical trials of aggressive medical management of cardiovascular risk factors late in CKD (e.g., during ESRD) have failed to significantly improve outcomes (Eknoyan et al. 2002; Locatelli et al. 2010; Palmer et al. 2013), further reinforce the importance of targeting intervention before irreversible remodeling is established. The addition of myocardial deformation imaging during mouse models of CKD can provide an additional cardiac parameter to monitor noninvasively during preclinical studies of therapeutic interventions.

## Conflict of Interest

None declared.
